# Molecular Detection of *Bartonella quintana* among Long-Tailed Macaques (*Macaca fascicularis*) in Thailand

**DOI:** 10.3390/pathogens10050629

**Published:** 2021-05-19

**Authors:** Wanat Sricharern, Supakarn Kaewchot, Phirabhat Saengsawang, Sarawan Kaewmongkol, Tawin Inpankaew

**Affiliations:** 1 Center for Agricultural Biotechnology, Kamphaengsaen Campus, Kasetsart University, Nakhonpathom 73140, Thailand; cvtwns@ku.ac.th; 2 Center of Excellence on Agricultural Biotechnology, Science and Technology Postgraduate Education and Research Development Office, Commission on Higher Education, Ministry of Education, Science, Research Innovation (AG-BIO/PERDO-CHE), Bangkok 10900, Thailand; 3 Department of Veterinary Technology, Faculty of Veterinary Technology, Kasetsart University, Bangkok 10900, Thailand; cvtswt@ku.ac.th; 4 Department of National Parks, Wildlife and Plant Conservation, Bangkok 10900, Thailand; supakarn_vet@hotmail.com; 5 Akkhraratchakumari Veterinary College, Walailak University, Nakhon Si Thammarat 80161, Thailand; phirabhat.s@gmail.com; 6 Department of Parasitology, Faculty of Veterinary Medicine, Kasetsart University, Bangkok 10900, Thailand

**Keywords:** *Bartonella quintana*, long-tailed macaque, *Macaca fascicularis*, Thailand

## Abstract

*Bartonella quintana* is a zoonotic pathogen with a worldwide distribution. Humans and non-human primates are considered to be natural reservoir hosts for *B. quintana.* However, information on the molecular epidemiology of this organism is very limited in regard to long-tailed macaques (*Macaca fascicularis*) in Thailand. Therefore, this study aimed to investigate the occurrence and genetic diversity of *Bartonella* spp. among long-tailed macaques in Thailand. In total, 856 blood samples were collected from long-tailed macaques in Thailand. All specimens were screened for *Bartonella* spp. using a polymerase chain reaction (PCR) assay targeting the 16S rRNA, *gltA* and *ftsZ* genes. All positive samples were further analyzed based on nucleotide sequencing, phylogenetic analysis and multiple sequence alignment analysis. Only one macaque showed a positive result in the PCR assays based on the 16S rRNA, *gltA* and *ftsZ* genes. Nucleotide sequencing and phylogenetic analysis revealed that the obtained sequences were closely related to *B. quintana* previously detected in non-human primates. Single-nucleotide polymorphisms (SNPs) were detected in the *gltA* and *ftsZ* gene sequences. This study revealed that long-tailed macaques in Thailand carried *B. quintana.* Despite the low infection rate detected, long-tailed macaques may be a reservoir of *B. quintana*.

## 1. Introduction

*Bartonella* spp. are Gram-negative, facultative and fastidious intracellular bacteria that can infect erythrocytes and endothelial cells. The genus *Bartonella* currently consists of at least 36 named and 17 Candidatus species [1,2]. Several species of *Bartonella* have been associated with human diseases such as *B. bacilliformis*, *B. henselae*, *B. vinsonii* subsp. *berkhoffii*, *B. elizabethae*, *B. alsatica* and *B. quintana*, which can cause numerous diseases, including Carrion’s disease, Oroya fever, cat scratch fever, endocarditis, neuroretinitis, trench fever and bacillary angiomatosis [1,2,3]. The disease manifestations range from subclinical and self-limiting infection to severe, life-threatening outcomes such as fever, headache, weight loss, muscle fatigue, partial paralysis, hallucinations and other neurological signs [2,3]. Transmission of *Bartonella* spp. mainly occurs via numerous arthropod vectors such as fleas, ticks, mites and lice [2,3].

*B. quintana* is an important human pathogen that causes a broad spectrum of diseases, including trench fever, chronic bacteremia, endocarditis, myocarditis, lymphadenopathy and bacillary angiomatosis, with severe disease possibly leading to death [4,5]. The human body louse (*Pediculus humanus humanus*) is known to be an arthropod vector of *B. quintana* in humans, especially among the homeless or people living in inadequate sanitary conditions [3,4]. Some studies have suggested that infections of *B. quintana* may be related to louse exposure [6]. Furthermore, vector transmission of *B. quintana* by *Pedicinus obtusus* lice—which are macaque-specific ectoparasites—has been described in rhesus macaques from China [7]. In addition, non-human primates are considered to be natural reservoir hosts of this human pathogen [3,4]. Detection of *B. quintana* has been reported in several species of non-human primates, including captive cynomolgus macaques (*Macaca fascicularis*) in the USA [8,9], captive rhesus macaques (*Macaca mulatta*) and cynomolgus macaques in China [6,7,10] and free-ranging Japanese macaques (*Macaca fuscata*) in Japan [11].

In Thailand, long-tailed macaques (*Macaca fascicularis*)—also called crab-eating macaques or cynomolgus macaques in laboratories—are the most frequently observed species of non-human primate [12,13]. There are many locations such as temples, forest parks and tourist attractions where these macaques reside close to human communities and share the environment with people. These macaques may act as reservoir hosts for many zoonotic pathogens that can be transmitted to people, including *Bartonella* spp. However, there has been a lack of information about the prevalence of *Bartonella* spp. infection among these monkeys. Therefore, the current study aimed to investigate the occurrence and genetic diversity of *Bartonella* spp. in free-ranging, wild, long-tailed macaques in Thailand.

## 2. Results

### 2.1. Molecular Identification of Bartonella spp.

Out of 856 samples screened for *Bartonella* DNA using a broad-range nested PCR assay based on the 16S rRNA gene, only one sample (0.1%) from a male macaque from Chonburi Province in eastern Thailand was positive. Since there was only one positive sample based on the 16S rRNA gene, we decided to screen all samples for *Bartonella* spp. infection again, this time using conventional PCR based on the *gltA* gene. The result revealed that the same sample was positive for *Bartonella* spp. Subsequently, this positive sample was subjected to PCR assays based on the *ftsZ* gene to confirm the species of *Bartonella* spp. The positive sample based on the 16S rRNA and *gltA* genes was also positive for PCR assay based on the *ftsZ* gene.

### 2.2. Nucleotide Sequencing

The results of nucleotide sequence alignment in the current study showed high identity with those reported in non-human primates from other countries. The *B. quintana* 16S rRNA sequence shared 100% identity with those of *B. quintana* isolated from Japanese macaques in Japan (GenBank accession no.: AP019773), rhesus macaques in China (GenBank accession no.: JQ314414) and a patient with endocarditis in Finland (GenBank accession no.: U28268). The *B. quintana gltA* sequence shared 99.35% identity with those of *B. quintana* isolated from Japanese macaques in Japan (GenBank accession no.: AP019773) and rhesus macaques in China (GenBank accession no.: CP003784 and JQ314417). The *B. quintana ftsZ* sequence shared 99.40% identity with sequences of *B. quintana* isolated from Japanese macaques in Japan (GenBank accession no.: AP019773) and rhesus macaques in China (GenBank accession no.: CP003784 and JQ314416).

### 2.3. Nucleotide Sequence Accession Numbers

The nucleotide sequences of the 16S rRNA, *gltA* and *ftsZ* genes of *B. quintana* from long-tailed macaques in Thailand were submitted to the GenBank database under the accession numbers MW301659, MW320725 and MW341113, respectively.

### 2.4. Phylogenetic Analysis

**Figure 1:**
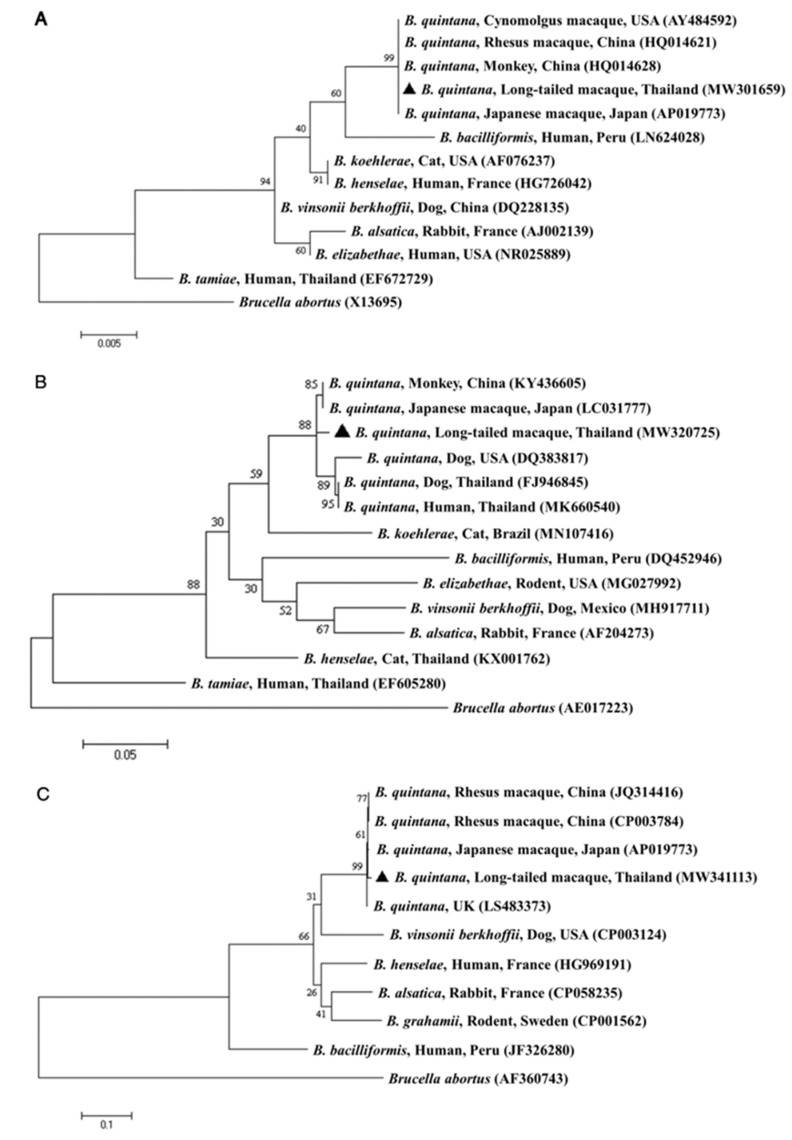
Phylogenetic trees of *Bartonella* spp. based on the 16S rRNA gene (**A**), *gltA* gene (**B**) and *ftsZ* gene (**C**). These trees were constructed using the maximum likelihood method based on the Kimura 2-parameter model with 1000 bootstrapping replications. Black triangles indicate sequences generated in the current study. GenBank accession numbers are shown in parentheses. *Brucella abortus* was used as the outgroup.

**Figure 2:**
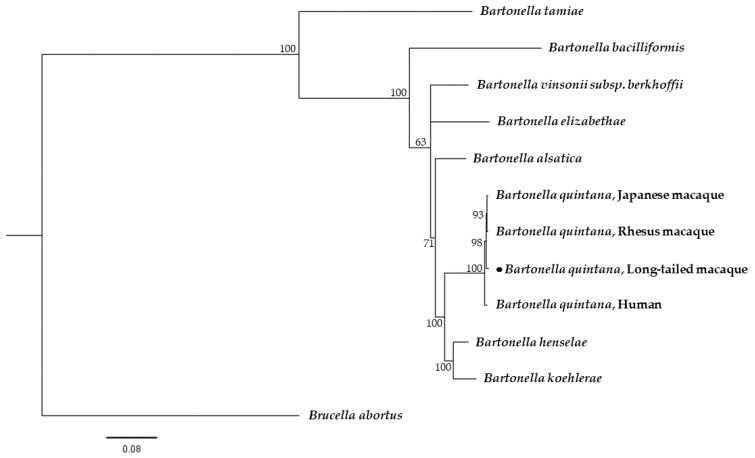
Phylogenetic tree of *Bartonella* spp. based on the combined 16S rRNA, *gltA* and *ftsZ* gene sequences (1721 bp). The tree was constructed using the Bayesian method based on the K2+G evolutionary model. Black dots indicate sequences generated in the current study. *Brucella abortus* was used as the outgroup.

The maximum likelihood phylogenetic analysis based on a fragment of the 16S rRNA gene demonstrated that the obtained sequence was closely related to the *B. quintana* detected in Japanese macaques from Japan, rhesus macaques from China and cynomolgus macaques from the USA. The sequence of the *gltA* gene obtained in this study was clustered along with other *B. quintana* sequences obtained from a patient in Thailand, monkeys in China, Japanese macaques in Japan and dogs in the USA and Thailand. In addition, the phylogenetic tree of the *ftsZ* gene revealed that the sequence in the current study was placed in the same clade as strains from Japanese macaques from Japan and rhesus macaques from China (Figure 1). In addition, the concatenated phylogenetic analysis based on the 16S rRNA, *gltA* and *ftsZ* genes showed that the *Bartonella* spp. sequence obtained from long-tailed macaques in this study was grouped in the same clade of *B. quintana* detected in Japanese macaques, rhesus macaques and humans previously deposited in GenBank, with a high clade support of 100, based on Bayesian inference with the Kimura two-parameter and gamma distribution (K2+G) evolutionary model (Figure 2).

### 2.5. Multiple Sequence Alignment Analysis

The partial sequences of the *gltA* and *ftsZ* genes of *B. quintana* obtained in the current study were compared to reference sequences from GenBank (GenBank accession numbers JQ314417 and JQ314416, respectively) using multiple sequence alignment analysis. Single-nucleotide polymorphisms (SNPs) were detected in two nucleotide positions of the *gltA* gene. The transitions C/T and A/G were found at positions 618 and 648 of the *gltA* gene fragment sequence, respectively. In the *ftsZ* gene fragment sequence, the transitions were observed in three nucleotide positions (1255, 1453 and 1725), whereas transversions were observed in two nucleotide positions (1530 and 1677). The data of the base substitutions are shown in Table 1.

## 3. Discussion

The current study demonstrated only one positive sample (1/856) of *B. quintana* infection in long-tailed macaques from Chonburi Province in eastern Thailand. The phylogenetic analysis based on the maximum likelihood method and Bayesian inference of the 16S rRNA, *gltA* and *ftsZ* genes confirmed that long-tailed macaques in Thailand carry *B. quintana*. To the authors’ knowledge, this is the first report of molecular detection of *B. quintana* among long-tailed macaques in Thailand. Previously, the detection of *B. quintana* in long-tailed macaques or cynomolgus macaques has been reported in captive long-tailed macaques from the USA [6,7] and China [10]. These findings suggested that long-tailed macaques might serve as reservoir hosts for *B. quintana.*

Higher prevalence rates of *B. quintana* infection among captive non-human primates have been reported in several studies, including 5.6% (2/36) in captive rhesus macaques from a biological research institute in China [8], 12% (37/308) in cynomolgus macaques and 18% (59/328) in rhesus macaques from primate centers in China [10] and 48.6% (34/70) in captive rhesus macaques from an animal facility in China [9]. On the other hand, the prevalence of *B. quintana* infection in wild non-human primates was reported in Japanese macaques (13.3%, 6/45) from Japan [11].

The associations between *B. quintana* infection and the gender or location of the hosts were not investigated in the current study due to the low number of positive samples. However, another study revealed no significant association between *B. quintana* infection and the gender of the hosts [9,10]. In addition, a previous study revealed that the prevalence of *B. quintana* infection in rhesus macaques was significantly higher than that in cynomolgus macaques, and that *B. quintana* infection rates in juvenile and young macaques were significantly higher than those for adult macaques [10].

The current study used molecular diagnosis based on PCR assays of the 16S rRNA, *gltA* and *ftsZ* genes to successfully identify *B. quintana*. However, the identification of *Bartonella* species based on the 16S rRNA gene was not considered satisfactory due to the high percentage of similarity between the sequences of this gene. Conversely, the *gltA* gene has been shown to be a reliable tool for distinguishing among *Bartonella* species [14,15]. In addition, another study confirmed that using one pair of primers enabled the comparison of partial *ftsZ* sequences among *Bartonella* species [16]. In addition to all three genes used in this study, other genes have also been reported in the classification of *Bartonella* strains, including the *groEL* and *ribC* genes and the ITS region [17,18,19].

Multiple sequence alignment analysis of the *gltA* and *ftsZ* gene sequences showed a number of SNPs compared to the sequences deposited in GenBank. Although only a few SNPs were identified due to the short DNA fragments, this result indicated that intraspecific variability exists within these genes of *B. quintana.*

The current study had certain limitations that should be improved in further studies. Firstly, the diagnosis of *B. quintana* based only on PCR assays identified the study’s lack of sensitivity, since the numbers of bacteremia found in asymptomatic non-human primates may be low [20]. Several studies have shown that cell culture techniques combined with PCR amplification could improve the chances of detection of *B. quintana* in the blood of non-human primates, such as in Japan, where *B. quintana* was isolated from Japanese macaques using chocolate agar cultivation together with PCR assay [11], and in China, where *B. quintana* was detected in rhesus macaque blood samples by isolation in sheep blood agar followed by PCR assay [8]. In addition to the aforementioned non-human primate studies, there are several previously published examples demonstrating the successful identification of *Bartonella* from blood samples using pre-enrichment culture in a *Bartonella* alpha-*Proteobacteria* growth medium (BAPGM) followed by PCR amplification, including 3.2% (16/500) in asymptomatic blood donors from Brazil [21], 27.0% (7/89) in companion animal veterinary personnel from Spain [22] and 31.3% (60/192) in stray dogs in Thailand [23]. Moreover, another study demonstrated 7.7% (20/261) *Bartonella* infection in acute febrile illness among patients in rural Thailand by using co-cultivation of blood samples with Vero-E6 cells and subsequent PCR analysis of the *gltA* gene [24]. Therefore, it is recommended to combine cell culture techniques with PCR assay for *Bartonella* spp. detection in future studies.

Another limitation was that the short partial DNA sequences of the 16S rRNA, *gltA* and *ftsZ* genes were used for sequence analysis, phylogenetic analysis and multiple sequence alignment analysis. To overcome this, it is recommended to use shotgun metagenomics sequencing or whole-genome sequencing (WGS) of this bacterium in order to better understand the pathogen.

The study of the epidemiology of *B. quintana* that may also infect humans justifies undertaking surveys of this bacterium among humans and companion animals such as dogs and cats in the same environment as non-human primates, since some studies have reported the detection of *B. quintana* in dogs from Bangkok, Thailand [23] and in cats from the USA [19]. For this reason, not only non-human primates but also dogs and cats may serve as reservoir hosts for *B. quintana* [25].

Besides human body lice, amplification of *B. quintana* DNA from cat fleas and macaque lice has been reported [1,7]. Unfortunately, arthropod vectors feeding on long-tailed macaques were not collected in the current study. Thus, further studies are needed to evaluate the role of arthropod vectors in the transmission cycle of *B. quintana* in macaques, other animals and humans.

## 4. Materials and Methods

### 4.1. Study Areas and Sample Collection

**Figure 3:**
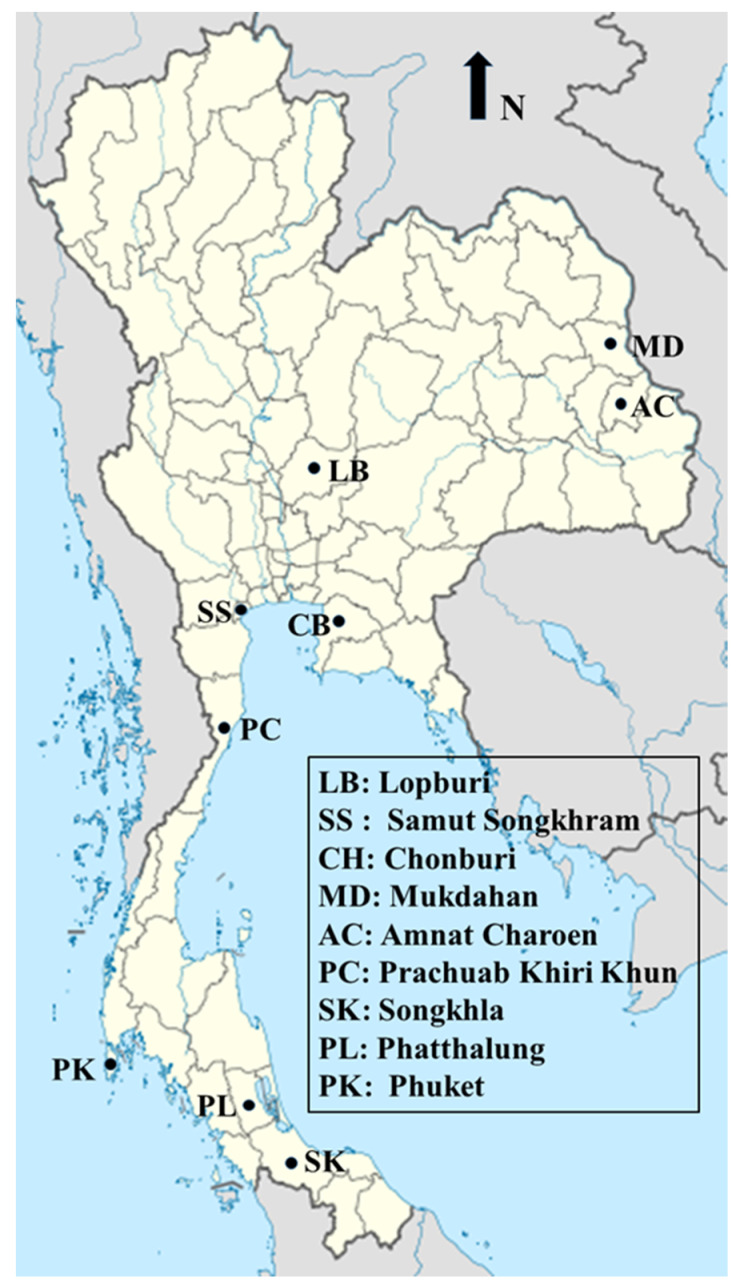
Map showing Thai provinces where blood samples from long-tailed macaques were collected (https://commons.wikimedia.org/wiki/File:Thailand_location_map.svg/, accessed on 11 March 2021).

The current study was conducted during 2016–2018 in 9 provinces of Thailand, consisting of Lopburi and Samut Songkhram in the central region; Chonburi in the eastern region; Mukdahan and Amnat Charoen in the northeastern region; Prachuab Khiri Khun in the western region; and Songkhla, Phatthalung and Phuket in the southern region (Table 2 and Figure 3). These locations represent areas where free-ranging wild long-tailed macaques live adjacent to human communities. In total, 856 blood samples were collected using convenience sampling from the long-tailed macaques by venipuncture. The samples were dispensed into an ethylenediaminetetraacetic acid (EDTA) tube and stored at −40 °C until used for subsequent DNA extraction.

### 4.2. Molecular Analysis

Genomic DNA was extracted from 200 µL of each whole blood sample using an E.Z.N.A.^®^ tissue DNA extraction kit (OMEGA Bio-tek Inc.; Norcross, GA, USA), according to the manufacturer’s instructions. All DNA samples were tested for *Bartonella* spp. infection. An existing, broad-range nested PCR protocol targeting the 16S rRNA gene was performed to amplify *Bartonella* spp. DNA using two sets of primers (Table 3). The 20-microliter PCR mixture for both primary and nested PCR samples contained 1× PCR buffer, 2 mM MgCl_2_, 0.2 mM dNTPs, 1 µM of each primer and 0.04 U/µL Taq DNA polymerase, using 2 µL of DNA extracted from the blood sample as a template.

The positive samples for the 16S rRNA gene were submitted to conventional PCR assays targeting another two genes, namely the citrate synthase gene (*gltA*) and the cell division protein gene (*ftsZ*). The data of the target genes and oligonucleotide sequences of these PCR protocols are listed in Table 3.

The PCR products were identified using electrophoresis on 1.2% (*w*/*v*) agarose-TAE gel and visualized with ultraviolet transillumination after staining the nucleic acid with GelStar^®^ (Cambrex Bio Science; Rockland, ME, USA). The positive samples with amplicons of the expected size were submitted for DNA purification. The sequences obtained from this study were submitted to BLAST analysis to determine similarities to other *Bartonella* spp. sequences previously deposited in the GenBank database.

### 4.3. Phylogenetic Analysis

Sequences obtained from positive samples in this study together with reference sequences downloaded from GenBank were aligned using the BioEdit program version 7.5.2. (https://bioedit.software.informer.com/, accessed on 9 April 2021). Phylogenetic analysis was constructed using the maximum likelihood method based on the Kimura 2-parameter model with 1000 bootstrap iterations using the MEGA-X software (http://www.megasoftware.net/, accessed on 9 April 2021). Additionally, a phylogenetic tree with the combined 16S rRNA, *gltA* and *ftsZ* genes was constructed based on the Bayesian inference phylogenetic analysis. The best evolutionary model was selected by the MEGA-X software (http://www.megasoftware.net/, accessed on 6 May 2021), under the Bayesian information criterion (BIC). The Bayesian inference phylogenetic analysis was performed with MrBayes 3.2.7 (http://nbisweden.github.io/MrBayes/download.html/, accessed on 6 May 2021).

### 4.4. Multiple Sequence Alignment Analysis

Multiple sequence alignment analyses of the DNA sequences obtained in this study and the sequences of *B. quintana* retrieved from the GenBank database were performed using the BioEdit program version 7.5.2 and the Clustal Omega program (http://www.ebi.ac.uk/Tools/msa/clustalo/, accessed on 9 April 2021) to find possible existing patterns of polymorphism in each gene.

## 5. Conclusions

To our knowledge, this is the first record of the occurrence of *B. quintana* among long-tailed macaques in Thailand. Despite the low infection rate observed, the results of the current study confirmed the natural infection of human pathogenic *B. quintana* among long-tailed macaques, from which it can be transmitted to other hosts including humans. Therefore, in addition to humans, it is important to continue investigating the prevalence of *B. quintana* in other species of non-human primates and companion animals living in the same environment. Importantly, further studies of the vectors and transmission routes must be undertaken to improve understanding of the transmission of this pathogen to humans and to prevent human cases of *B. quintana* infection.

## Figures and Tables

**Table 1:** Multiple sequence alignment analysis showing SNPs found within partial sequences of *B. quintana* for the *gltA* and *ftsZ* genes compared with reference sequence.

Gene	GenBank Accession Number	Homology (%)	Nucleotide at Position				
*gltA*			618	648			
Reference	JQ314417	99.35%	C	A			
	MW320725		T	G			
*ftsZ*			1255	1453	1530	1677	1725
Reference	JQ314416	99.40%	A	C	C	T	G
	MW341113		G	T	A	G	A

Nucleotide positions are numbered according to the reference *gltA* partial sequence (GenBank accession number: JQ314417), with the first nucleotide as position 1 and with reference to the *ftsZ* partial sequence (GenBank accession number: JQ314416), with the first nucleotide as position 1.

**Table 2:** Location of sample collection and numbers of samples.

Region	Province	Male	Female	Total Number
Central	Lopburi	81	112	193
	Samut Songkhram	18	7	25
East	Chonburi	253	47	300
Northeast	Mukdahan	30	0	30
	Amnat Charoen	70	29	99
West	Prachuab Khiri Khun	28	38	66
South	Songkhla	34	5	39
	Phatthalung	30	0	30
	Phuket	67	7	74
	Total	611	245	856

**Table 3:** Oligonucleotides sequences, target genes and amplicon size (bp) used in nested PCR assay targeting the 16S rRNA gene and conventional PCR assays targeting the *gltA* and *ftsZ* genes for *Bartonella quintana* detection.

Gene	Primer	Oligonucleotide Sequences	Amplicon Size (bp)	Ref.
*16S rRNA*	V1-F(a)	5′-AGAGTTTGATCCTGGCTCAG-3′	1400	[26]
	V9-R(a)	5′-GNTACCTTGTTACGACTT-3′		
	V3-F(b)	5′-ACTCCTACGGGAGGCAGCAG-3′	700	
	V6-R(b)	5′-CGACAGCCATGCANCACCT-3′		
*gltA*	BhCS.781p	5′-GGGGACCAGCTCATGGTGG-3′	390	[14]
	BhCS.1137n	5′-AATGCAAAAAGAACAGTAAACA-3′		
*ftsZ*	BaftsZF	5′-GCTAATCGTATTCGCGAAGAA-3′	900	[16]
	BaftsZR	5′-GCTGGTATTTCCAAYTGATCT-3′		

(a) = primers for primary PCR; (b) = primers for secondary PCR; Ref. = References; bp = base pair.

## Data Availability

Data are contained within the article.
